# A Comprehensive Systems Biological Study of Autophagy-Apoptosis Crosstalk during Endoplasmic Reticulum Stress

**DOI:** 10.1155/2015/319589

**Published:** 2015-04-23

**Authors:** Marianna Holczer, Margita Márton, Anita Kurucz, Gábor Bánhegyi, Orsolya Kapuy

**Affiliations:** Department of Medical Chemistry, Molecular Biology and Pathobiochemistry, Semmelweis University, Tűzoltó Utca 37-47, Budapest 1094, Hungary

## Abstract

One of the most important tasks of a living organism is to maintain its genetic integrity with respect to stress. Endoplasmic reticulum (ER) has a crucial role in sensing cellular homeostasis by controlling metabolism, proteostasis, and several signaling processes. ER stressors can induce autophagy-dependent survival; however excessive level of stress results in apoptotic cell death. Although many molecular components of these networks have already been discovered, the analysis of the dynamical features of the regulatory network of life-or-death decision is still lacking. Our goal was to incorporate both theoretical and molecular biological techniques to explore the autophagy-apoptosis crosstalk under ER stress. Using various levels of different ER stressors we confirmed that the control network always generated an evidently detectable autophagy-dependent threshold for apoptosis activation. We explored the features of this threshold by introducing both autophagy activators and inhibitors, and transient treatment with excessive level of ER stressor was also performed. Our experimental data were also supported by a stochastic approach. Our analysis suggests that even if the switch-like characteristic of apoptosis activation is hardly seen on population level the double negative feedback loop between autophagy and apoptosis inducers introduces bistability in the control network.

## 1. Introduction

The maintenance of intrinsic homeostasis in a multicellular organism is mainly dependent on the ability of cells to take precise actions with respect to external and internal stimuli (such as nutrient availability, inflammatory mediators, and growth factors) [1, 2]. The generated response mechanism (e.g., cell growth and division and cell death) has to provide an accurate decision by taking precise actions to avoid any “misunderstanding” and its fatal consequences. The comprehensive molecular networks and their system-level crosstalks have an essential role in achieving the correct characteristic of the answer. These crosstalks guarantee both the robustness and the proper dynamical feature of the regulatory system in response to various signals. The existence of different crosstalks between traditionally considered separate signaling pathways has been got into highlights recently [3].

Endoplasmic reticulum (ER) is a eukaryotic organelle that has a crucial role in sensing cellular homeostasis and generating suitable signals and responses [4]. ER has major functions in synthesizing, folding, and packaging secreted and membrane proteins of the cell [5, 6]. ER has a key role in metabolism (such as lipid biosynthesis and carbohydrate metabolism) and several signaling processes, too [7]. For these integrated roles of ER a special redox homeostasis and a high luminal Ca^2+^ environment are required [8, 9].

An imbalanced luminal ER homeostasis might result in the activation of various ER stress response mechanisms [4, 7, 10, 11]. The precise balance between production and consumption of folded proteins is tightly regulated by a complex network of signaling pathways, called unfolded protein response (UPR) [12, 13]. Accumulation of incorrectly folded proteins immediately turns on UPR. The signaling pathways of UPR have three well-defined transducers activated upon ER stress, called IRE1 (inositol requiring 1), PERK (PKR-like ER kinase), and ATF6 (activating transcription factor 6), respectively [13, 14]. All three components are ER-resident transmembrane proteins and are kept inactive by the same Grp78/BIP protein. While activation of both IRE1 and ATF6 promotes transcription of UPR target genes (such as chaperones), PERK-controlled pathway leads to the general inhibition of protein translation [14, 15].

Corresponding to harmful ER stress the response mechanism immediately accelerates the formation of autophagosomes. This observation is confirmed by increasing autophagic function with respect to ER stress [16, 17]. Autophagy is an evolutionary conserved cellular digestive process whereby cytosolic contents are sequestered in double membrane vesicles (so-called autophagosomes) and delivered to the lysosome to form an autophagolysosome. The digested components get recycled by the cell; therefore it is claimed that autophagy has a crucial protective role after ER stress [2, 18, 19]. It was also suggested that autophagy promotes survival with respect to UPR-induced ER stress by “self-eating” of damaged elements [2, 18, 19].

However severe ER stress can result in apoptosis-dependent cell death [16, 20, 21]. The key function of apoptosis is to remove aberrant or damaged cells, but it also has an important role in eliminating cells during embryonic development and immune system maturation [22, 23]. Apoptotic cell death guarantees a controlled abolition of the selected cell by cellular shrinkage, mitochondrial permeabilization, chromatin condensation, and DNA fragmentation. Apoptosis can be induced by either extra- or intracellular stimuli triggering the extrinsic or intrinsic pathways, respectively [24]. The intrinsic pathway can be activated by the wide range of cellular stress signals (such as DNA damage and ER stress) resulting in mitochondrial outer membrane permeabilization and cytochrome *c* release. Extrinsic apoptosis gets initiated by death-receptors [25]. The most commonly used drugs to perturb ER homeostasis are thapsigargin (TG) and tunicamycin (TM). TG disrupts the calcium storage of ER by blocking calcium reuptake into the ER lumen via SERCA, while TM inhibits N-linked glycosylation in the ER [7, 14]. Traditionally both ER stressors were supposed to induce autophagy but Ganley et al. have pointed out that this is not exactly the case [26]. They claimed that TG did not affect the proper autophagosome formation, instead of the fusion of autophagosome with the endocytotic system which was blocked. Therefore TG is not an autophagy inducer but rather an inhibitor of the process resulting in a latent accumulation of autophagosomes [26]. However both TM and TG are great inducers of apoptotic cell death [27–29]. Since dithiothreitol (DTT) is a strong reducing agent, it is also used to generate ER stress by disturbing oxidative protein folding in the ER lumen [30].

Although the networks of both autophagy and apoptosis are complex, more and more results confirm that these two pathways are not independent of each other; they are linked at various levels generating a so-called crosstalk with even more comprehensive regulatory networks between them [31, 32]. While autophagy has a prosurvival effect, apoptosis induces cell death; therefore this crosstalk seems to have an essential role in a well-balanced cellular response with respect to various stress signals (e.g., ER stress, nutrient deprivation) [32].

New experimental data have revealed the existence of a crosstalk between the survival and killing pathways on the level of caspases and autophagy-inductor Beclin-1 regulated by Bcl2 [33, 34]. Although the details of this mechanism are still unknown, a small model of the underlying control network is suggested by our lab [35]. This model claims that autophagy has a sigmoid activity peak even at low level of cellular stress, while apoptosis remains inactive. However, excessive level of cellular stress results in an irreversible switch-like activation of apoptosis inducer and autophagy inducer gets inactive [35]. We suggested that the effect of crosstalk element and the double negative feedback loop between autophagy and apoptosis inducers have a crucial role in guaranteeing the proper decision-making between life and death [36]. Later Xu et al. confirmed experimentally that autophagy induction in response to stress is uniformly unimodal, while apoptotic process gets activated in bimodal fashion [37]. They claimed that apoptosis has an all-or-none characteristic with a sigmoid characteristic of its signal response curve. Using a remarkable single cell analysis cells were treated with various stressors to confirm their results (such as staurosporine and rapamycin). Tunicamycin treatment has shown that although its effect was similar to the other drugs, its influence was much slower and weaker on autophagy induction as compared to the addition of staurosporine [37]. Recently we have also shown that inhibition of mTOR pathway increases cell viability via autophagy induction during ER stress using a novel autophagy activator, metyrapone [29].

In the present study a systematic analysis was performed to explore even further the autophagy-apoptosis crosstalk with respect to ER stress. We show by using both molecular and theoretical biological methods that autophagy always generates an evidently distinguishable threshold for apoptosis activation. The importance of the strength of autophagy was studied by both autophagy activator and inhibitor. To test the irreversible dynamical profile of apoptosis induction, transient treatments were done with excessive level of ER stress. We claim that all the three well-known ER stressors (TG, TM, and DTT) acting by different mechanisms have similar effects in various human cell lines (such as HepG2 and HEK293). In a parallel way a stochastic model of the simplified control network was used to get a more realistic picture about the decision-making process between life and death.

## 2. Materials and Methods

### 2.1. Materials

Metyrapone (Sigma-Aldrich, M2696), thapsigargin (Sigma-Aldrich, T9033), rapamycin (Sigma-Aldrich, R0395), tunicamycin (Sigma-Aldrich, T7765), DTT (Merck, K39637174 905), and 3-methyladenine (Sigma-Aldrich, M9281) were purchased. All other chemicals were of reagent grade.

### 2.2. Cell Culture and Maintenance

As model system, human liver carcinoma (HepG2) and human embryonic kidney (HEK293) cell lines were used. It was maintained in DMEM (Life Technologies, 41965039) medium supplemented with 10% fetal bovine serum (Life Technologies, 10500064) and 1% antibiotics/antimycotics (Life Technologies, 15240062). Culture dishes and cell treatment plates were kept in a humidified incubator at 37°C in 95% air and 5% CO_2_.

### 2.3. SDS-PAGE and Western Blot Analysis

Cells were harvested and lysed with 20 mM Tris, 135 mM NaCl, 10% glycerol, 1% NP40, and pH 6.8. Protein content of cell lysates was measured using Pierce BCA Protein Assay (Thermo Scientific, 23225). During each procedure equal amounts of protein were used. SDS-PAGE was done by using Hoefer miniVE (Amersham). Proteins were transferred onto Millipore 0.45 *μ*M PVDF membrane. Immunoblotting was performed using TBS Tween (0.1%), containing 5% nonfat dry milk for blocking membrane and for antibody solutions. Loading was controlled by developing membranes for GAPDH or dyed with Ponceau S in each experiment. The following antibodies were applied: anti-LC3B (SantaCruz, sc-16755), anti-caspase-3 (SantaCruz, sc-7272), anti-PARP (Cell Signaling, 9542S), anti-p62 (Cell Signaling, 5114S), and anti-GAPDH (Santa Cruz, 6C5) and HRP conjugated secondary antibodies (SantaCruz, sc-2020, and Cell Signaling, 7074S, 7076S).

### 2.4. Statistics

For densitometry analysis western blot data were acquired using ImageQuant 5.2 software. The relative band densities were shown and normalized to an appropriate GAPDH band used as reference protein (see Supplementary Information available online at http://dx.doi.org/10.1155/2015/319589). Results are presented as mean values ± S.D. and were compared using ANOVA with Tukey's multiple comparison post hoc test. Asterisks indicate statistically significant difference from the appropriate control: ^∗^ *P* < 0.05; ^∗∗^ *P* < 0.01.

### 2.5. Cell Viability Assays

Cell viability was detected using CellTiter-Blue assay (Promega, G8080). Cells were grown and treated on 96-well plates and were incubated with resazurin for 2 h at 37°C. Absorbance was measured at 620 nm and expressed in arbitrary unit, being proportional to cell toxicity. For each of these experiments at least three parallel measurements were carried out.

### 2.6. Annexin Staining

Apoptotic and necrotic cells were detected by using fluorescence microscopy and Annexin-V-FLUOS staining kit (Roche, 11988549001). Cells were grown and treated on 96-well plates and were treated with the kit according to the manufacturer's instructions. Cells with green fluorescence were considered as apoptotic, while those with red or both red and green (orange) fluorescence were considered as necrotic. In each experiment a minimum of 1000 cells was counted.

### 2.7. Mathematical Modeling

The regulatory network was translated into a set of nonlinear ordinary differential equations (ODEs) and analyzed using the techniques of dynamical system theory [38–40]. For details see Supplementary Information. Dynamical simulations were carried out using the program* XPPAUT*, which is freely available from http://www.math.pitt.edu/~bard/xpp/xpp.html [39, 40]. We provide the XPP codes that can be used to generate all the figures in the paper.

## 3. Results

### 3.1. ER Stress Induced Apoptotic Cell Death Is Always Preceded by Autophagy-Dependent Survival

**Figure 1:**
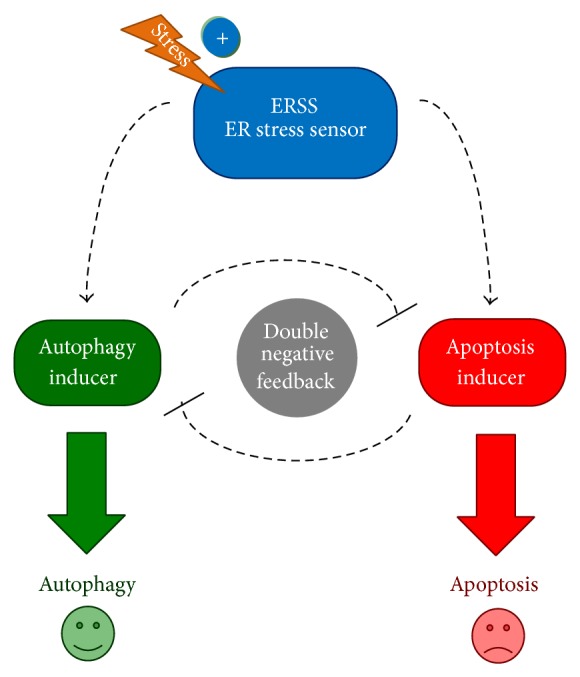
The schematic model of autophagy-apoptosis crosstalk during ER stress. The autophagy inducer, the apoptosis inducer, and the ER stress sensor (ERSS) are denoted by isolated green, red, and blue boxes, respectively. Dashed line shows how the molecules can influence each other, while blocked end lines denote inhibition.

Different studies have shown that autophagy is activated as an initial response to ER stress followed by apoptotic cell death. Recently, we have shown using mathematical modeling approach that sequentially activation of autophagy and apoptosis depends upon the crosstalk between the signaling pathways involved in the activation of these cellular processes [35]. This study also predicted that the activation of autophagy and apoptosis happens in a mutually exclusive manner and is independent of ER stress inducers (Figure 1). To test the model further, we treated both HepG2 and HEK cells with different ER stressors (TG, TM, and DTT) and studied the temporal activation of autophagy and cell death inducers.

**Figure 2:**
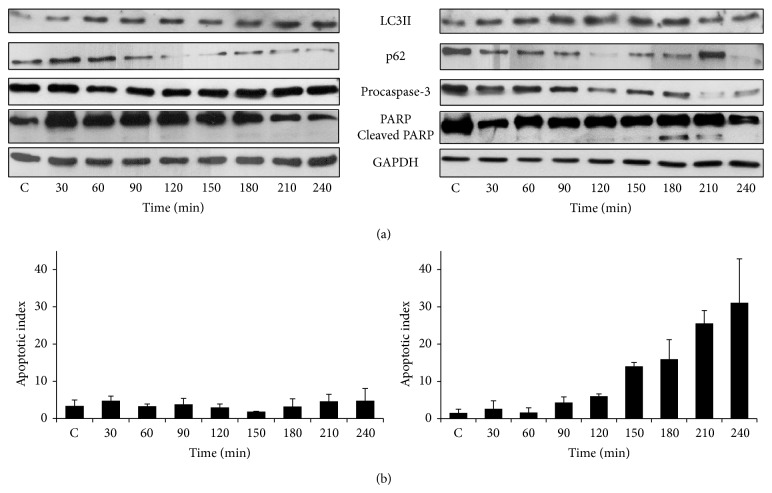
The time course profile of cell treatment with respect to low and high levels of TM. HepG2 cells were treated with (left panel) low (1 *μ*M) and (right panel) high (100 *μ*M) level of TM. (a) The autophagy (LC3II, p62) and apoptosis markers (procaspase-3, cleaved PARP) were followed in time by immunoblotting. (b) The percentage of apoptotic cells was calculated by Annexin-V-FLUOS kit (errors bars represent standard deviation).

In order to choose the concentration of ER stressor that can be used to study different cellular responses, we carried out cell viability assay at different concentrations of stressors (data not shown). We identified both low and high concentrations of TM (low, 1 *μ*M, high, 100 *μ*M), TG (low, 0.1 *μ*M, high, 50 *μ*M), and DTT (low, 1 mM, high, 10 mM) for HepG2 cells that could preserve and diminish cell viability, respectively. To explore the kinetic profile of autophagy and cell death mechanisms the well-known autophagy (such as LC3II and p62) and apoptosis (e.g., procaspase-3, cleaved PARP) markers were followed in time by immunoblotting. In the presence of low concentration of TM, both the formation of LC3II and a decrease in the level of p62 were observed indicating an effective autophagic response even at low level of the stressor (Figure 2(a), left panel, and Figure S1). Meanwhile apoptotic cell death markers were not induced (i.e., the level of procaspase-3 remained high and PARP cleavage was not detected). At higher concentration of TM, a transient activation of autophagic markers was observed between 30 and 180 min (Figure 2(a), right panel, and Figure S2). This was followed by the induction of apoptosis markers at 210 min. We also confirmed the result obtained by immunoblotting by counting the number of cells undergoing apoptosis in the total population by using Annexin-V-FLUOS kit. It can be seen that the treatment with the lower concentration of TM resulted in the induction of apoptosis in only 3–5% of cells, which was not increased with respect to time (Figure 2(b), left panel). However, in the presence of higher concentration of TM, the percentage of cells undergoing apoptosis increased to 20–30% after 180 minutes (Figure 2(b), right panel).

**Figure 3:**
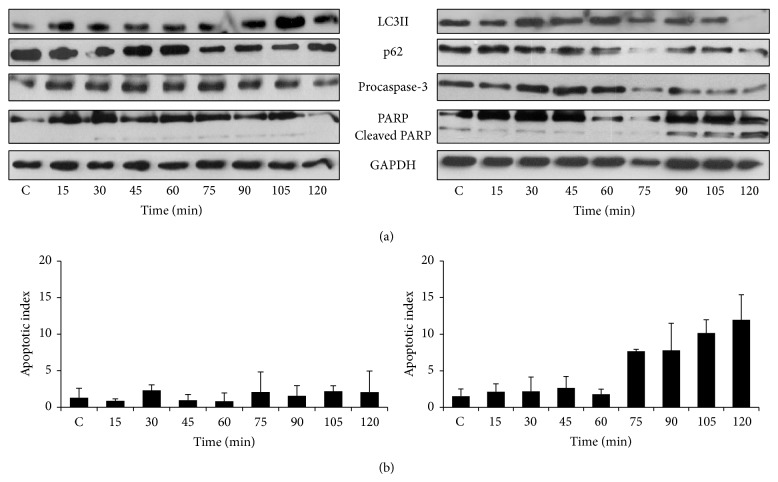
The time course profile of cell treatment with respect to low and high levels of DTT. HepG2 cells were treated with (left panel) low (1 mM) and (right panel) high (10 mM) level of DTT. (a) The autophagy (LC3II, p62) and apoptosis markers (procaspase-3, cleaved PARP) were followed in time by immunoblotting. (b) The percentage of apoptotic cells was calculated by Annexin-V-FLUOS kit (errors bars represent standard deviation).

This immediately raises the question whether the observed effects are ER stressor specific. Therefore, we tested the cellular response to other stressors such as DTT and TG. Interestingly, treatment of cells with DTT (Figures 3, S3, and S4) and TG (Figure S5) also showed that autophagy markers appear under both lower and higher concentrations but disappear with appearance of apoptosis markers at the higher concentration of these drugs. The only difference was the time of appearance of apoptosis markers (and disappearance of autophagy markers). It can be seen that the higher concentration of DTT caused a rapid raise in percentage of cells undergoing apoptosis after 75 min (Figure 3(a), right panel and Figure S4) whereas this occurred at 180 min in the case of TM (Figure 2(a), right panel). Ganley et al. have claimed that TG blocks autophagy, while our data shows that this could be the result of only transient activation of autophagy at higher concentration of TG (Figure S5). We got similar results when HEK cells were treated with different ER stressors (Figure S6). These results suggest that autophagy is activated even at high concentration of various ER stressors and this could provide a window of opportunity for the cells to survive. However, in the continuous presence of stress, cell death mechanism gets activated and disables cell survival mechanism such as autophagy.

### 3.2. Apoptotic Cell Death Gradually Increases in the Population with respect to ER Stress

We have previously shown by mathematical modeling that under ER stress apoptosis activation is stepwise whereas autophagy inducer increases gradually. Although in experiments we observe some evidence for rapid shift in apoptosis markers and a stepwise increase in the percentage of cells undergoing apoptosis (Figures 2 and 3 and S5), it is not very obvious from these population measurements whether apoptosis inducer is activated rapidly in comparison to autophagy inducer. However, a single cell behavior simulated by the deterministic model can be different from the population behavior. We demonstrate this difference by developing a simple stochastic model of the molecular mechanism as shown in Figure 1 (for the detailed description about the model see Supplementary Information). Stochastic fluctuations can act at different levels and bring about intracell and cell-cell variations giving a good description about cell population behavior with respect to ER stress.

**Figure 4:**
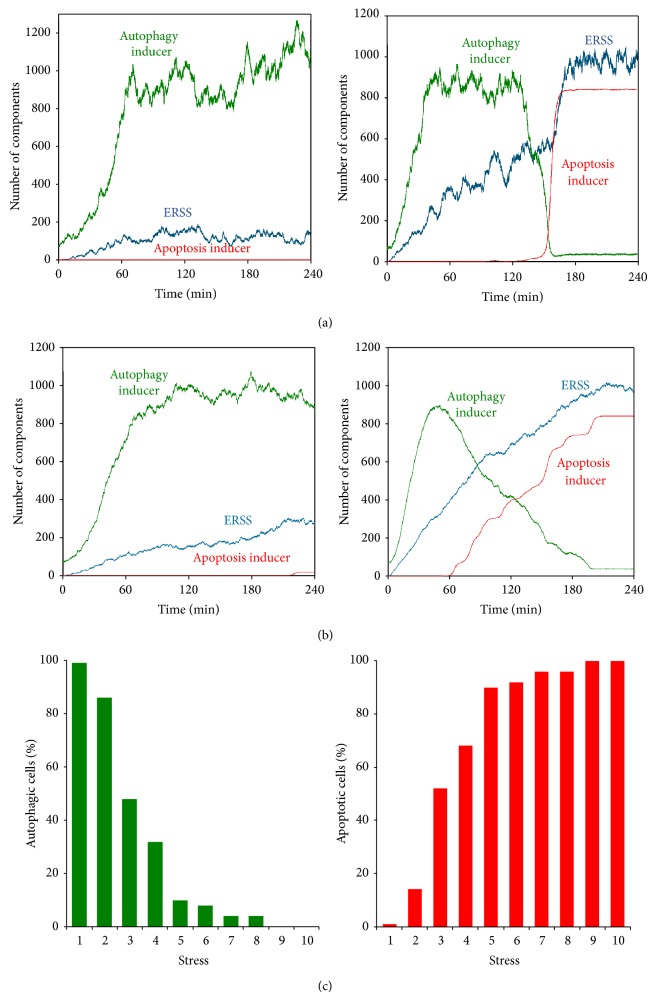
Stochastic simulation of cell population with respect to ER stress. (a) Single cell treatment with (left panel) low (S0 = 1) and (right panel) high (S0 = 5) level of ER stress. (b) The probability distributions of autophagy and apoptosis were studied during various treatments by doing 50 independent simulations. The averaged behavior of a cell population is computed with respect to (left panel) low (S0 = 1) and (right panel) high (S0 = 5) level of ER stress. (c) Probability distributions of the strength of ER stressor in cell populations. 50 single cell simulations were done at any different levels of ER stressor from S0 = 1 to 10. The percentage of autophagic (left panel) and apoptotic (right panel) cells was checked after 240 min long treatment.

Figure 4(a) shows the simulated behavior of the single cell in the presence of noise. At low concentration of ER stressor (Figure 4(a), left panel) the autophagy inducer gets activated gradually, while apoptosis inducer remained inactive. On the other hand, at high concentration of ER stressor, the autophagy inducer gets activated transiently and this is followed by the rapid activation of apoptosis inducer (Figure 4(a), right panel). It can be noted that the stepwise activation of apoptosis inducer can still be observed in the presence of noise at the single cell level.

In order to simulate the cell population behavior, we carried out 50 simulations and obtained the average levels of autophagy and apoptosis inducers.  Figure 4(b), left panel, shows that in the presence of lower concentration of ER stressor the average level of autophagy inducers increased gradually after 30 minutes. Interestingly, at high level of ER stress the transient peak of autophagy inducer is followed by a gradual increase in the average level of apoptosis inducer (Figure 4(b), right panel). Individual cells activate apoptosis in a step-like manner; however the time of activation differs. This suggests that the population measurements might average out all the individual cell behavior and make the apoptosis inducers activation a gradual process as observed in our experiments. Therefore, we cannot rule out the stepwise activation of apoptosis inducers at the single cell level (see the deterministic version of our model in Figures S7(A)-(B)). Further, the quick activation kinetics also suggests that the apoptosis activation would show a switch-like characteristic with respect to different stress levels and its activation depends upon the stress level crossing a critical threshold value [35]. We studied the cell population behavior by computing the percentage of cell undergoing apoptosis with respect to different stress values. It can be seen that at low stress levels (for *S* < 3) the majority of cells only activated the autophagy inducer while at high stress levels the majority of cells only activated apoptosis inducer (Figure 4(c)). At intermediate levels of stress (3 < *S* < 5) both populations coexisted. This suggests that the stochasticity of ER stress strength in the cell population influences the threshold value of stress required to activate the apoptosis, which leads to gradual increase in the percentage of cells undergoing apoptosis with respect to different stress levels. Therefore, within the cell population, we observed a time distribution for apoptosis inducer activation in the presence of stress (Figure S7(C)).

### 3.3. Autophagy Has a Crucial Role in Determining the Threshold for Apoptosis Activation under ER Stress

The threshold value of stress required to activate apoptosis inducer depends upon the regulatory mechanism that controls (or contributes to) its switch-like activation. We study how the crosstalk between autophagy and apoptosis inducers controls the threshold value of stress required to induce apoptotic switch. We used both autophagy activator and inhibitor to study their effect on the activation of apoptosis inducer.

**Figure 5:**
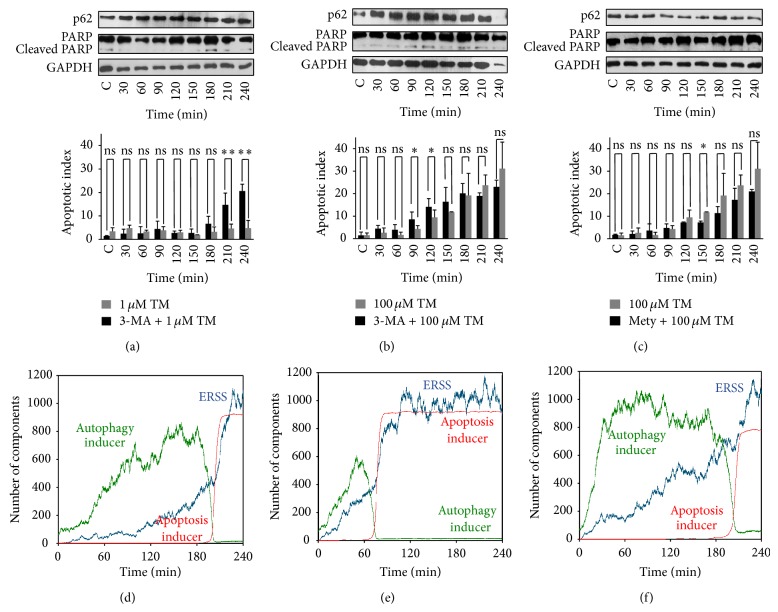
The role of autophagy to determine the activation threshold for apoptosis. HepG2 cells were pretreated with autophagy inhibitor ((a) and (b) 1 mM 3-methyladenine for 2 hours) or activator ((c) 100 *μ*M metyrapone for 2 hours) before low ((a) 1 *μ*M) or high ((b) and (c) 100 *μ*M) level of 4-hour long TM treatment and the main autophagy (p62) and apoptosis markers (cleaved PARP) were followed in time by immunoblotting ((a), (b), and (c)). The percentage of apoptotic cells was followed by Annexin dye (errors bars represent standard deviation; asterisks indicate statistically significant difference from the control: ^∗^ *P* < 0.05; ^∗∗^ *P* < 0.01) ((d), (e), and (f)). ((d)-(e)-(f)) The mathematical simulation of single cell treatment. ((d) and (e)) The activation term of autophagy inducer reduced to kaau' = 0.2, while S0 = 1 on (d) and 5 on (e). (f) The activation term of autophagy inducer increased to kaau' = 0.7 and S0 = 5.

Cells were pretreated with 3-methyladenine (3-MA) for 2 hours, which is a well-known inhibitor of autophagy by blocking the formation of autophagosomes. Further, these cells were treated with either low (1 *μ*M) or high (100 *μ*M) concentration of TM and their effect on the activation of autophagy and apoptosis was followed in time. Interestingly, we observed the activation of apoptosis (PARP was cleaved and apoptosis index increased) after 210 min long treatment even at low level of TM (Figures 5(a), 5(d) and S8(A)), while its activation was not observed in the absence of pretreatment with autophagy inhibitor (Figure 2(a)). These results suggested that the threshold for apoptosis activation was pushed to lower stress levels when autophagy was downregulated. Interestingly, apoptosis was activated earlier at 120 minutes when high level of TM was combined with 3-MA (Figures 5(b), 5(e) and S8(B)).

Metyrapone is an autophagy activator [41], since it is shown to increase the efficiency of autophagic process via downregulation of mTOR pathway. The pretreatment of cells with metyrapone before the addition of TM delayed the activation of apoptosis by more than 1 hr (Figures 5(c), 5(f) and S8(C)). Similar effects were observed by using TG or DTT (data not shown). These data show that autophagy has a crucial role in determining the activation threshold of apoptosis under ER stress. Its activation can shift the activation threshold of apoptosis to higher stress levels while its inhibition shifts it to lower stress levels.

### 3.4. Irreversible Characteristic of Apoptosis Activation under ER Stress

Our results indicate that autophagy inducers can block or delay apoptosis induction. However, it is also known that apoptosis inducers also inhibit autophagy-dependent survival by cleaving some of the key proteins involved in autophagy activation (such as Beclin-1) [32]. We have shown in our previous work that this mutual antagonism between autophagy and apoptosis inducers can make the apoptosis activation an irreversible bistable switch [35]. Irreversible switch refers to point-of-no-return; that is, once the cell is engaged in apoptosis induction (autophagy inactivation) it never returns back to its previous self-healing state even if the level of stress decreases. This bistable characteristic of the control network is essential to avoid the proliferation of severely damaged cells.

**Figure 6:**
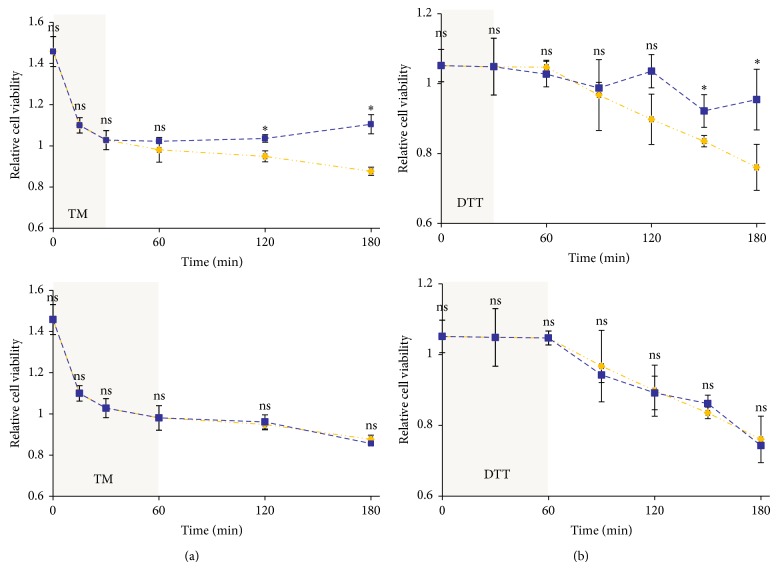
(a) TM (100 *μ*M) was washed out after (upper panel) 30 min and (lower panel) 60 min treatment. (b) DTT (10 mM) was washed out after (upper panel) 30 min and (lower panel) 60 min treatment (errors bars represent standard deviation; asterisks indicate statistically significant difference from the control: ^∗^ *P* < 0.05).

To verify experimentally the irreversibility of apoptosis induction with respect to high level of ER stress we performed inhibitor washout experiments in which cells were treated with high level of ER stressor for certain time interval and the inhibitor was washed out to study the effect on cell viability. Since the binding affinity of TG to SERCA is so high, the washout of this stressor is almost impossible. Therefore, the experiment was done by transient addition of excessive level of TM or DTT. Using cell viability assay it can be observed that washing out the high concentration of TM (100 *μ*M) after 30 min prevented the further decrease in viability (Figure 6(a), upper panel). However, the washout of TM after 60 min failed to prevent the decrease in viability. Similar profile was observed with DTT treatment (Figure 6(a), lower panel). The washout of high level of DTT (10 mM) after 30 min was able to prevent further decrease in cell viability (Figure 6(b), upper panel), while 60 min long treatment with excessive level of ER stressor was fatal for the cell population (Figure 6(b), lower panel).

**Figure 7:**
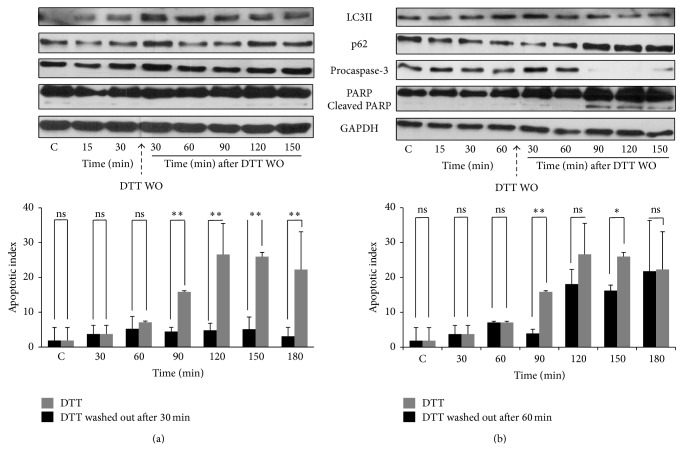
The irreversibility of apoptotic induction is tested by transient treatment with high level of ER stressor. HepG2 cells were treated with high concentration of DTT and washed out after (left panel) 30 min and (right panel) 60 min (a). The autophagy (LC3II, p62) and apoptosis markers (procaspase-3, cleaved PARP) were followed in time by immunoblotting. (b) The percentage of apoptotic cells was calculated by Annexin-V-FLUOS kit (errors bars represent standard deviation; asterisks indicate statistically significant difference from the control: ^∗^ *P* < 0.05; ^∗∗^ *P* < 0.01).

To study events at molecular level during inhibitor washout experiments we also detected the autophagy and apoptosis markers by immunoblotting.  Figure 7 shows the result of washing out DTT after 30 or 60 min treatment. When 10 mM DTT was washed out after 30 min, apoptosis marker was not detected (e.g., no PARP cleavage) (Figures 7(a) and S9). Autophagy marker was detected with DTT treatment and with washout it decreased slightly after 120 min. However, when the washout was performed after 60 min, the autophagy markers disappear after 90 min of washout; meanwhile PARP cleavage was observed suggesting the activation of apoptotic cell death (Figures 7(b) and S10). We also calculated the apoptotic index in the washout experiment (Figure 7). The washout of cells after longer treatment with DTT increased the percentage of apoptotic cells to ~20%, while after shorter treatment it was only ~5%.

**Figure 8:**
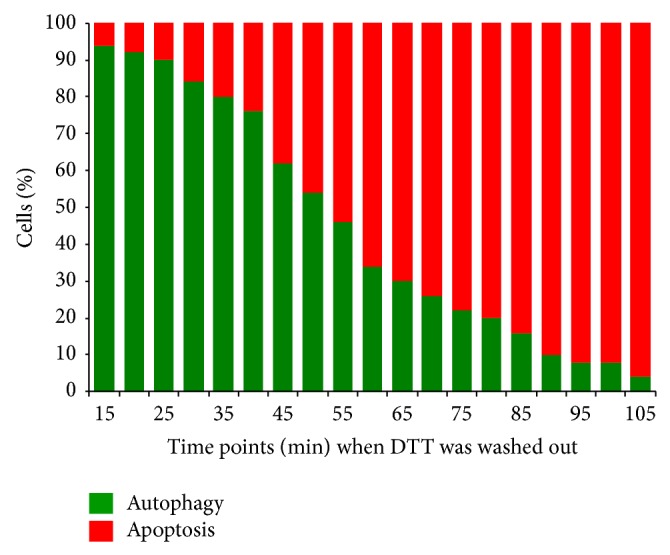
Analyzing the effect of transient treatment with mathematical method. 50 single cell simulations were done at all different time points when the ER stressor was washed out from 15 to 105 min (S0 = 10). The reversibility of apoptosis induction was checked after 120 min of depletion of ER stressor.

We mimicked the effect of washout of ER stressor performed at different time points (from 15 min to 105) on cell population behavior by using our mathematical model (Figure 8). The activity of autophagy/apoptosis inducer was calculated after 120 min of ER stressor washout. Most cells underwent autophagy when the high level of ER stressor was washed out after short treatment, while the washout after long treatment results in apoptosis on 95% of the population. At intermediate times, the choice between life and death is influenced by stochasticity. The percentage of cells committed to apoptosis gradually increases between 30 min and 90 min. These results suggest that even though ER stress level is well above the threshold for apoptosis activation, cells commit suicide only when cell death process irreversible overcomes cell survival mechanism.

## 4. Discussion

The cellular decision-making process between survival and death is driven by a complex regulatory network. The first crucial task of the control system is to decipher the magnitude and duration of stress. In this work, we studied the dynamics of activation of both autophagy-dependent survival and apoptotic self-killing mechanisms in response to different ER stressors (such as TG, TM, and DTT). Our results indicate that although the ER stressors disturb the cellular organelle differently (i.e., TG blocks SERCA, DTT modifies the redox homeostasis in the ER lumen, and TM inhibits N-linked glycosylation) the control system of survival/death decision has to be the same (Figures 2, 3, S5, and S6). We showed here that low level of ER stress always induces autophagy. However at a high stress level the autophagy is transiently activated as an initial response followed by apoptosis activation; meanwhile autophagy gets rapidly inhibited. We suggest that crosstalk between autophagy and apoptosis pathways is a generic design principle involved in controlling apoptosis activation to various ER stressors.

We observed a gradual increase in both autophagy and apoptosis inducers at the population level. However, we demonstrated by stochastic modeling that the apoptosis activation can be a rapid process once cell is engaged to it but it happens at different times in individual cells (Figures 4 and S7(C)). We claim that the average behavior of cell population can render apoptosis induction to a gradual process (Figure 4(b)). Such differences have been previously reported in treatment of cells with TRAIL [42]. Interestingly, the induction of autophagy has a sigmoid characteristic in both single cell and cell population simulations supposing that a stepwise activation characteristic for survival mechanism is not required (Figure 4).

Rapid activation of apoptosis suggests that self-killing mechanism gets activated in a switch-like manner when ER stress level crosses a threshold value. To reproduce the experimentally observed phenomena in our simple model, this is due to the assumption of multistep modifications of apoptosis inducers, which are known to give rise to switch-like behavior (see Supplementary Information). However, different mechanisms have already been proposed within extrinsic and intrinsic apoptosis pathways that can give rise to switch-like activation of apoptosis. Therefore, further study is required to understand the molecular mechanism involved in the switch-like activation of apoptosis inducer under ER stress.

We also showed that autophagy-dependent survival has an important antiapoptotic effect in the presence of various ER stressors and achieves this by directly influencing the threshold for apoptosis activation (Figure 5). We observed that inhibition of autophagy with 3-methyladenine leads to activation of apoptosis even at low stress levels, while hyperactivation of autophagy with metyrapone pretreatment delays the activation of apoptosis at severe ER stress. These results undoubtedly suggest that the activation threshold of self-killing mechanism is sensitive to autophagy. Antiapoptotic role of autophagy under ER stress suggests that apoptosis activation also depends on autophagy inactivation. Since autophagy gets rapidly downregulated when apoptosis switches on (Figures 2, 3, S5, and S6) we claim that a mutual antagonism between autophagy and apoptosis inducers has a crucial role in controlling the decision-making process with respect to various ER stressors.

We observed that the threshold for the activation of apoptosis is influenced by the stochasticity of cell population (Figures 4 and 5). Therefore, individual cells have different activation threshold, which in turn influences the time of activation of apoptosis. We think this is due to the critical slowing down phenomenon near the activation threshold of apoptosis. Therefore, apoptosis gets activated with a time lag in the cells with activation threshold closer to the actual stress level while time lag is decreased in cells with activation threshold further away from the actual stress level. However the sigmoid characteristic of autophagy induction is the same in both single cell and cell population simulations. These results suggest that experiments studying cell population (i.e., immunoblotting) are sufficient to give a good description about the dynamical characteristic of autophagy induction with respect to ER stress. However, single cell experiments would be essential to carry out for the analysis of apoptosis induction in the future.

We have shown previously that mutual antagonism between apoptosis and autophagy inducers could make the system bistable but does not make the apoptosis activation irreversible (see Figure 5 in [35]). We also showed that its irreversibility depends on extra positive feedback loops such as those between Bcl2 (crosstalk element) and caspases (apoptosis inducer). In the present study, we performed ER inhibitor washout experiments with TM and DTT to study the irreversible activation of apoptosis (Figure 6). Our study showed that earlier washout of high level of stressor prevented further drop in cell viability while late washout diminished the viability further similar to continuous treatment with excessive level of ER stressor. The immunoblot data show that autophagy remains active at short treatment, while late washout cannot block apoptosis induction (Figure 7). These results confirm the irreversible characteristic of self-killing mechanism when the treatment reaches a critical level in the cell. However the time of commitment to irreversible activation of apoptosis varies between individual cells and depends on the antagonism between autophagy and apoptosis (Figure 8). We suppose that extra crosstalk between crosstalk element and apoptosis inducer is essential for irreversibility, but identification of this regulatory loop needs experimental studies in the future. Moreover, the finding that the activation of the prosurvival factor autophagy seems to be an inevitable consequence of any kind of ER stresses suggests a therapeutic value for preconditioning with ER stress in a variety of human diseases.

## Supplementary Material

Supplementary Material contains a detailed description about the stochastic model used for supporting the experimental data by computational simulations. This material also involves the specification of the complete set of elements and parameters of the model and the simulation code is also attached. Second half of the electronic data contains the densitometry analysis of each western blot results depicted in the main text and the experiments done with different cell lines and stressors are also shown.

## Abbreviations

ER: Endoplasmic reticulum
TM: Tunicamycin
TG: Thapsigargin
DTT: Dithiothreitol
UPR: Unfolded protein response.
