# Supplementary material for: A Comprehensive Systems Biological Study of Autophagy-Apoptosis Crosstalk during Endoplasmic Reticulum Stress
Source: Biomed Res Int. 2015 Apr 23;2015:319589. doi: 10.1155/2015/319589 (PMC4423012; doi:10.1155/2015/319589)
Supplement: Supplementary file 1 — Supplementary Material contains a detailed description about the stochastic model used for supporting the experimental data by computational simulations. This material also involves the specification of the complete set of elements and parameters of the model and the simulation code is also attached. Second half of the electronic data contains the densitometry analysis of each western blot results depicted in the main text and the experiments done with different cell lines and stressors are also shown. [file 319589.f1.pdf]

Supplementary Material

“A comprehensive systems biological study of autophagy-apoptosis crosstalk during endoplasmic reticulum stress”

The model description

A simple stochastic model was developed to study the qualitative behavior of the regulatory system. For stochastic simulations Chemical Langevin Equation (CLE) is used, which is a stochastic differential equation driven by a multidimensional Wiener process. This method is widely used in applied mathematics, economics and physics (e.g. to describe random walk). Many textbooks and papers give us a detailed description about the Chemical Langevin Equation method used for this process (Gillespie, 200; Andrews et al., 2009). Each differential equation is supplemented with a noise term, which is proportional to the square root of the individual rate expression. To apply this stochastic method the following criteria has to be fulfilled:

1. well-mixed solution for the reactions: mixing always occurs faster than the relevant biochemical reaction (Sotiropoulos et al., 2008),
2. presence of both fast and slow reactions (Salis and Kaznessis, 2005),
3. the participating elements are abundant enough to assume their concentrations change continuously, but stochastic fluctuations still play a major role (Mélykúti et al., 2010).

According to the requirements the components involved in the ER stress response are grouped into three different modules, such as ER stress sensor (ERSS), autophagy (Auta) and apoptosis (Apoa) inducers, respectively. ERSS promotes both Auta and Apoa upon ER stress. Each component exists either in active or inactive form. To take into account that autophagy activation first overtakes apoptosis induction with respect to ER stress, an activation cascade

is assumed for apoptosis. In our simple model Apoa has four forms (Apoa1, Apoa2, Apoa3 and Apoa4, respectively) and each can be in an active or an inactive state, but only Apoa4 is able to induce apoptosis. The double negative feedback between Auta and Apoa shares mutual antagonistic relationship.

In the wiring diagram the autophagy inducer, the apoptosis inducer and the ER stress sensor (ERS) are denoted by isolated green, red and blue boxes, respectively. The active forms of the components are grouped in dark-colored boxes, while light-colored boxes denote the inactive forms. Solid arrows represent biochemical reactions; dashed line shows how the components can influence each other.

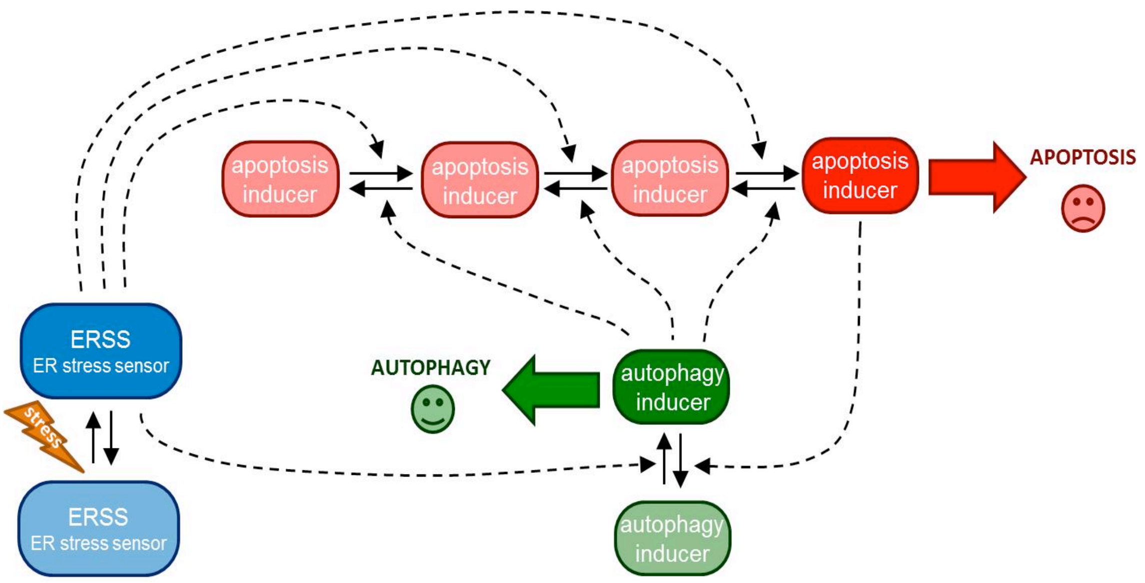

The detailed description about the elements, constants and biochemical reaction are found in the following tables. The above-mentioned code was computed numerical using *XPP-AUT*. Since the three module of the model involved huge amount of components therefore a large (around 1000) copy numbers were used for each element. Parameter values are estimated according to the experimental data. By our definition the cell could enter autophagy or apoptosis if the copy numbers of autophagy or apoptosis inducer reached 750 at a given time point during computer simulations.

The detailed description of the elements of the theoretical models

	**description - components**
**ERSS**	the active form of ER stress sensor
**ERSST**	the total ER stress sensor
**Auta**	the active form of autophagy inducer
**Auti**	the inactive from of autophagy inducer
**Autt**	the total autophagy inducer
**Apoa**	one of the inactive forms of apoptosis inducer
**Apoa2**	one of the inactive forms of apoptosis inducer
**Apoa3**	one of the inactive forms of apoptosis inducer
**Apoa4**	the active form of apoptosis inducer
**Apot**	the total apoptosis inducer
**S**	Stress

The detailed description of the constants of the theoretical models

	**description - constants**
**kaers'**	stress-dependent activation constant of ERSS
**kaers"**	apoptosis inducer-dependent activation constant of ERSS
**kiers**	background inactivation constant of ERSS
**kaap'**	ERSS-dependent activation constant of apoptosis inducer
**kiap**	background inactivation constant of apoptosis inducer
**kiap'**	autophagy inducer-dependent inactivation constant of apoptosis inducer
**kaau**	background activation constant of autophagy inducer
**kaau'**	ERSS-dependent activation constant of autophagy inducer
**kiau**	background inactivation constant of autophagy inducer
**kiau'**	apoptosis inducer-dependent inactivation constant of autophagy inducer

The detailed description of biochemical reactions in the model

	**biochemical reactions**
**activation term of ERSS**	kaers'*S + kaers"*Apoa4
**inactivation term of ERSS**	kiers
**activation term of AUTA**	kaau + kaau’*ERSS
**inactivation term of AUTA**	kiau + kiau'*Apoa4
**activation term of APOA**	kaap'*ERSS
**inactivation term of APOA**	kiap + kiap'*Auta

The XPP code for stochastic simulations

```
# this version is changed the noise term for each single reaction process
init S0=5, S=0, ERS=0.04, AUTA=71.11, APOA2=0.16, APOA3=0, APOA4=0, TIME=0

# generating a series of normally distributed random numbers
wiener wers
wiener wauta
wiener wapoa2
wiener wapoa3
wiener wapoa4

S0'= 0
S' = lev*(V*S0 - S)

ERSS' = (kaers'/V*S + kaers"/V*Apoa4)*(V*ERSST-ERSS) - kiers*ERSS + eers*wers*sqrt(ERSS)

Auta' = (kaau + kaau'/V*ERSS)*(V*Autt-Auta) - (kiau + kiau'/V*Apoa4)*Auta + eauta*wauta*sqrt(Auta)
Auti = V*Autt - Auta

Vaapo = kaap'/V*ERSS
Viapo = kiap + kiap'/V*Auta

Apoa1 = V*Apot - Apoa4 - Apoa2 - Apoa3
Apoa2' = Viapo*Apoa3 - Vaapo*Apoa2 + Vaapo*Apoa - Viapo*Apoa2
Apoa3' = Viapo*Apoa4 - Vaapo*Apoa3 + Vaapo*Apoa2 - Viapo*Apoa3
Apoa4' = Vaapo*Apoa3 - Viapo*Apoa4 + eapoa4*wapoa4*sqrt(Apoa4)

time' = 1
global +1 {time-depriv} {S0=0}

p V=1000, lev=0.5, depriv=500
p kaers'=0.001, kaers"=0.1, kiers=0.001, ERSST=1
p Apot=1, kaap'=1.5, kiap=0.05, kiap'=5
p Autt=1, kaau=0.0001, kaau'=0.5, kiau=0.001, kiau'=15
p eers=1, eauta=1, eapoa2=0, eapoa3=0, eapoa4=0

done
```

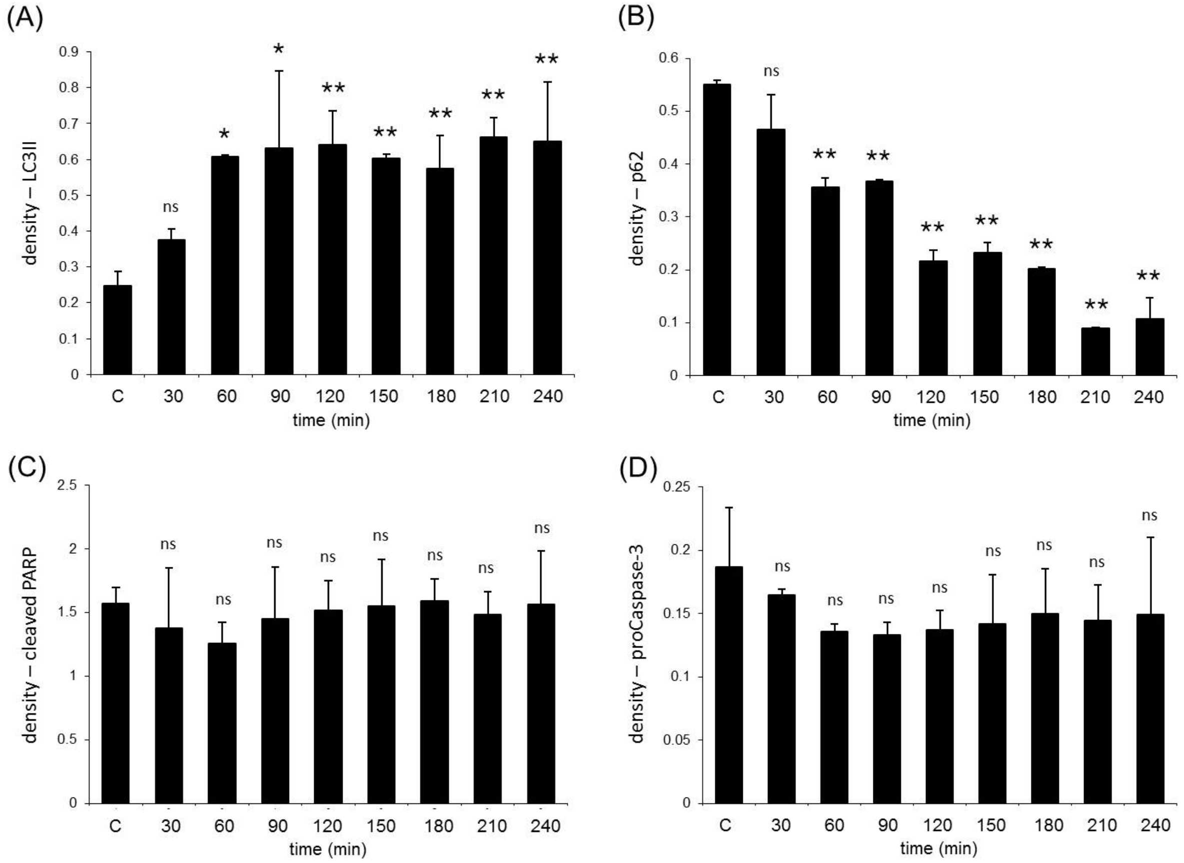

Figure S1. The time course profile of cell treatment with respect to low level of TM (1 μM). Densitometry data represent the intensity of **(A)** LC3II, **(B)** p62, **(C)** cleaved PARP and **(D)** procaspase-3 bands. These band intensities are shown in arbitrary units after normalization of each band to its appropriate GAPDH band, separately (errors bars represent standard deviation, asterisks indicate statistically significant difference from the control: ⋆ - $p < 0.05$; ⋆⋆ - $p < 0.01$).

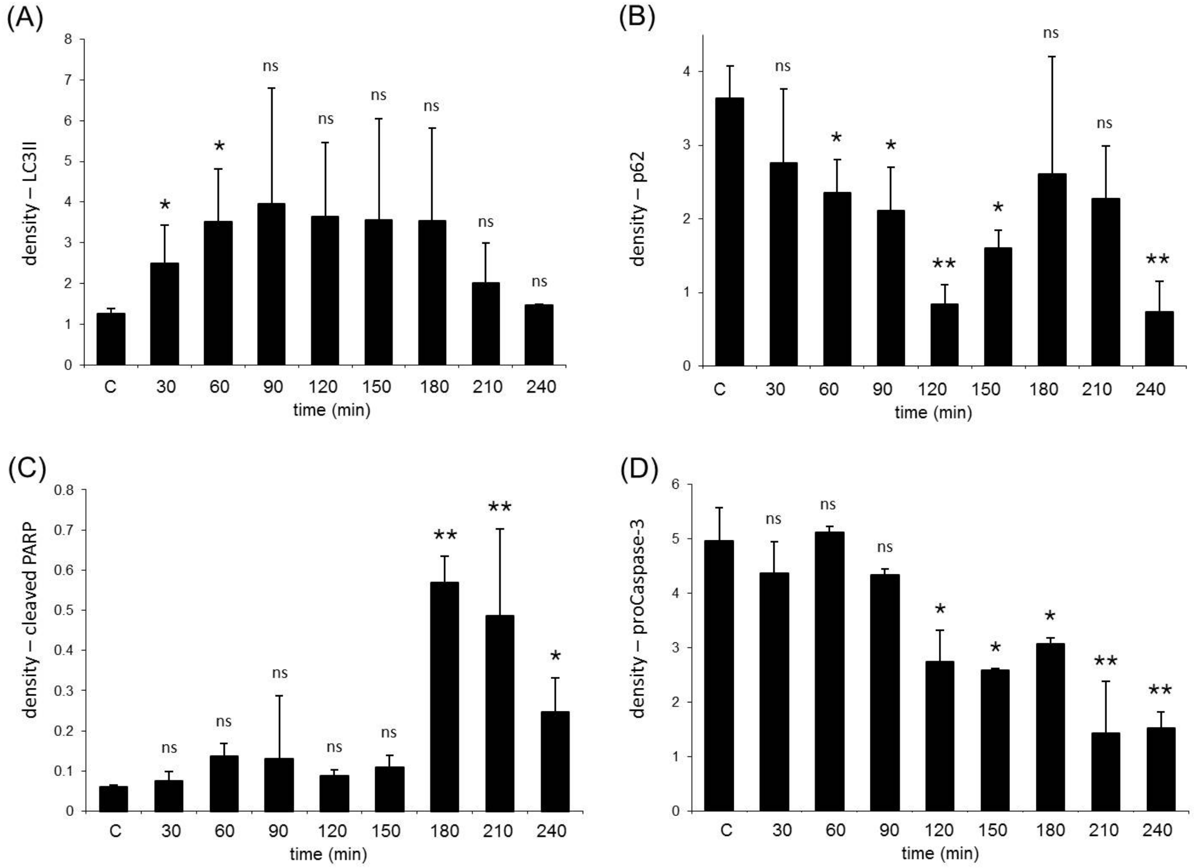

Figure S2. The time course profile of cell treatment with respect to high level of TM (100 μM). Densitometry data represent the intensity of **(A)** LC3II, **(B)** p62, **(C)** cleaved PARP and **(D)** procaspase-3 bands. These band intensities are shown in arbitrary units after normalization of each band to its appropriate GAPDH band, separately (errors bars represent standard deviation, asterisks indicate statistically significant difference from the control: ⋆ - $p < 0.05$; ⋆⋆ - $p < 0.01$).

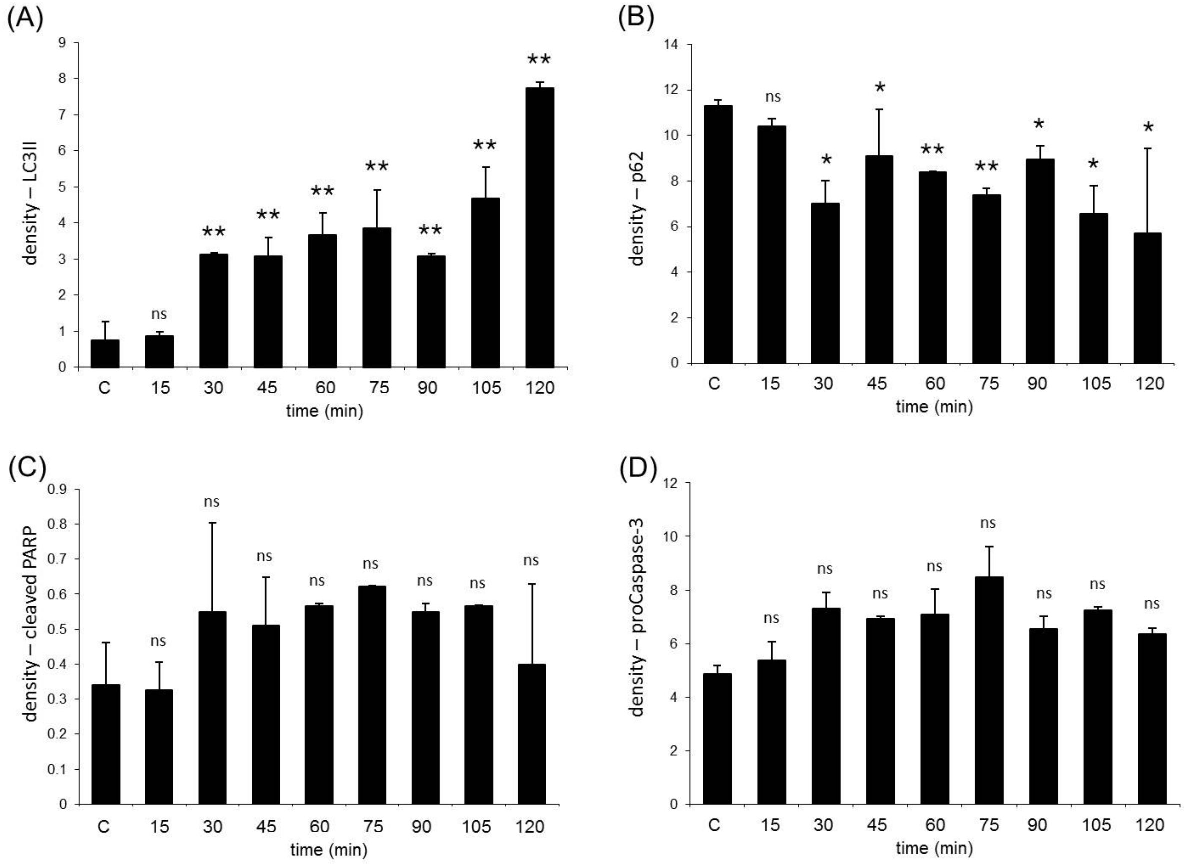

Figure S3. The time course profile of cell treatment with respect to low level of DTT (1 mM). Densitometry data represent the intensity of **(A)** LC3II, **(B)** p62, **(C)** cleaved PARP and **(D)** procaspase-3 bands. These band intensities are shown in arbitrary units after normalization of each band to its appropriate GAPDH band, separately (errors bars represent standard deviation, asterisks indicate statistically significant difference from the control: ⋆ - $p < 0.05$; ⋆⋆ - $p < 0.01$).

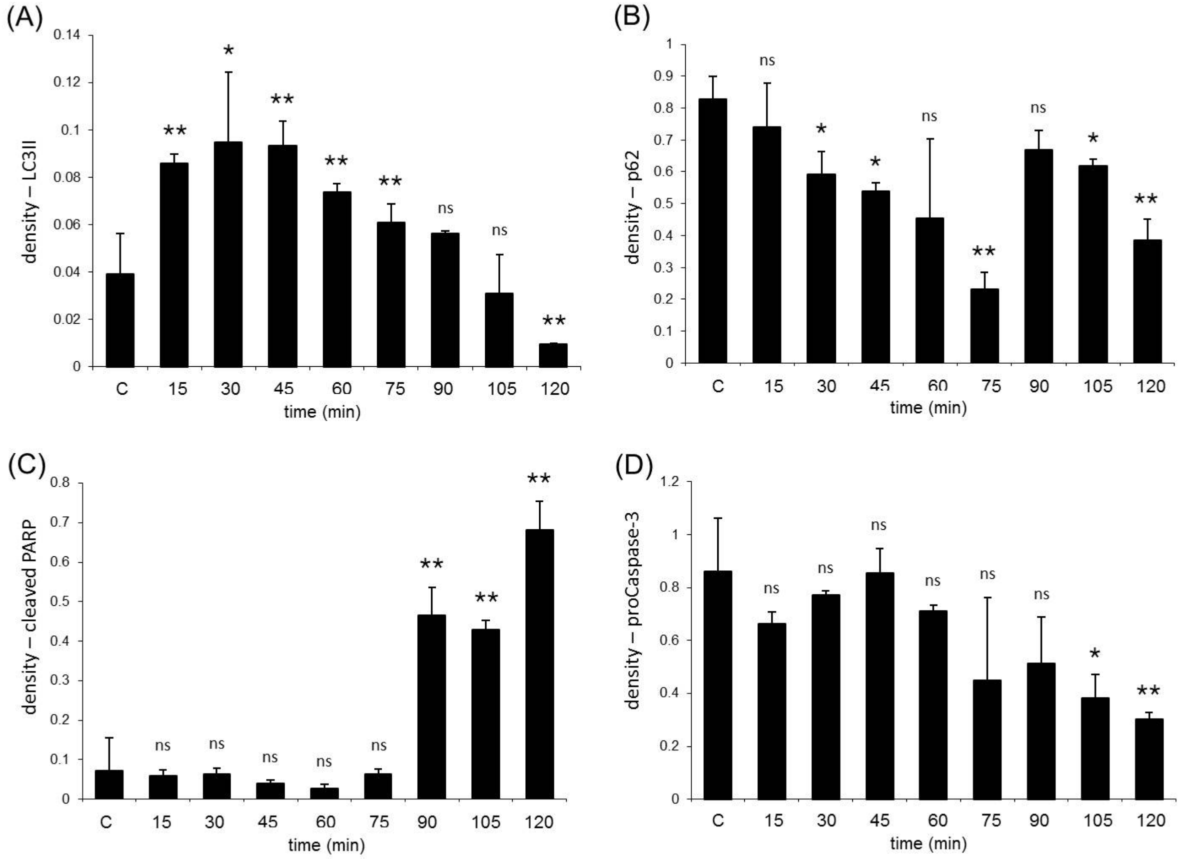

Figure S4. The time course profile of cell treatment with respect to high level of DTT (10 mM). Densitometry data represent the intensity of **(A)** LC3II, **(B)** p62, **(C)** cleaved PARP and **(D)** procaspase-3 bands. These band intensities are shown in arbitrary units after normalization of each band to its appropriate GAPDH band, separately (errors bars represent standard deviation, asterisks indicate statistically significant difference from the control: ⋆ - $p < 0.05$; ⋆⋆ - $p < 0.01$).

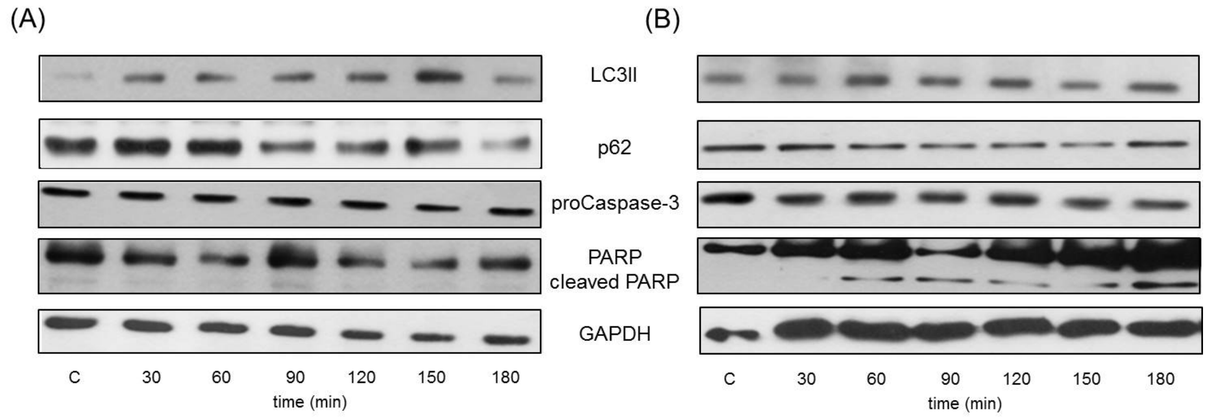

Figure S5. The time course profile of cell treatment with respect to low and high levels of TG. HepG2 cells were treated with **(A)** low (0.1 μM) and **(B)** high (50 μM) level of TG. The autophagy (LC3II, p62) and apoptosis markers (procaspase-3, cleaved PARP) were followed in time by immunoblotting.

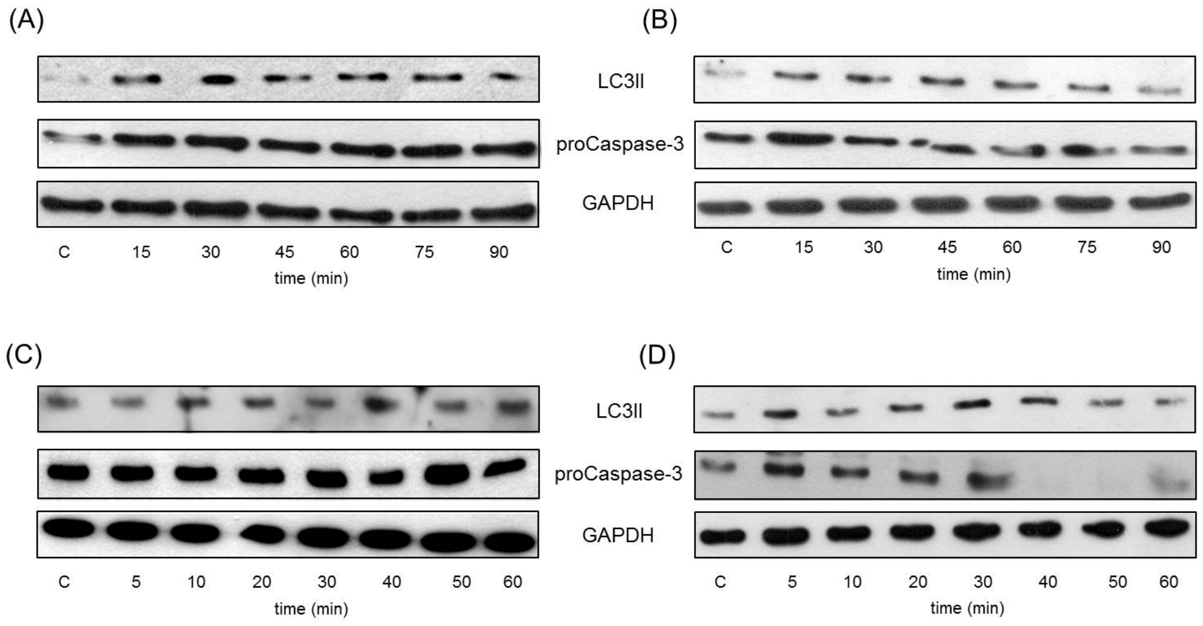

Figure S6. The time course profile of HEK cell treatment with various ER stressors. HEK293 cells were treated with **(A)** low (0.1 μM) and **(B)** high (10 μM) level of TG. HEK293 cells were treated with **(C)** low (1 mM) and **(D)** high (5 mM) level of DTT. The autophagy (LC3II) and apoptosis markers (procaspase-3) were followed in time by immunoblotting.

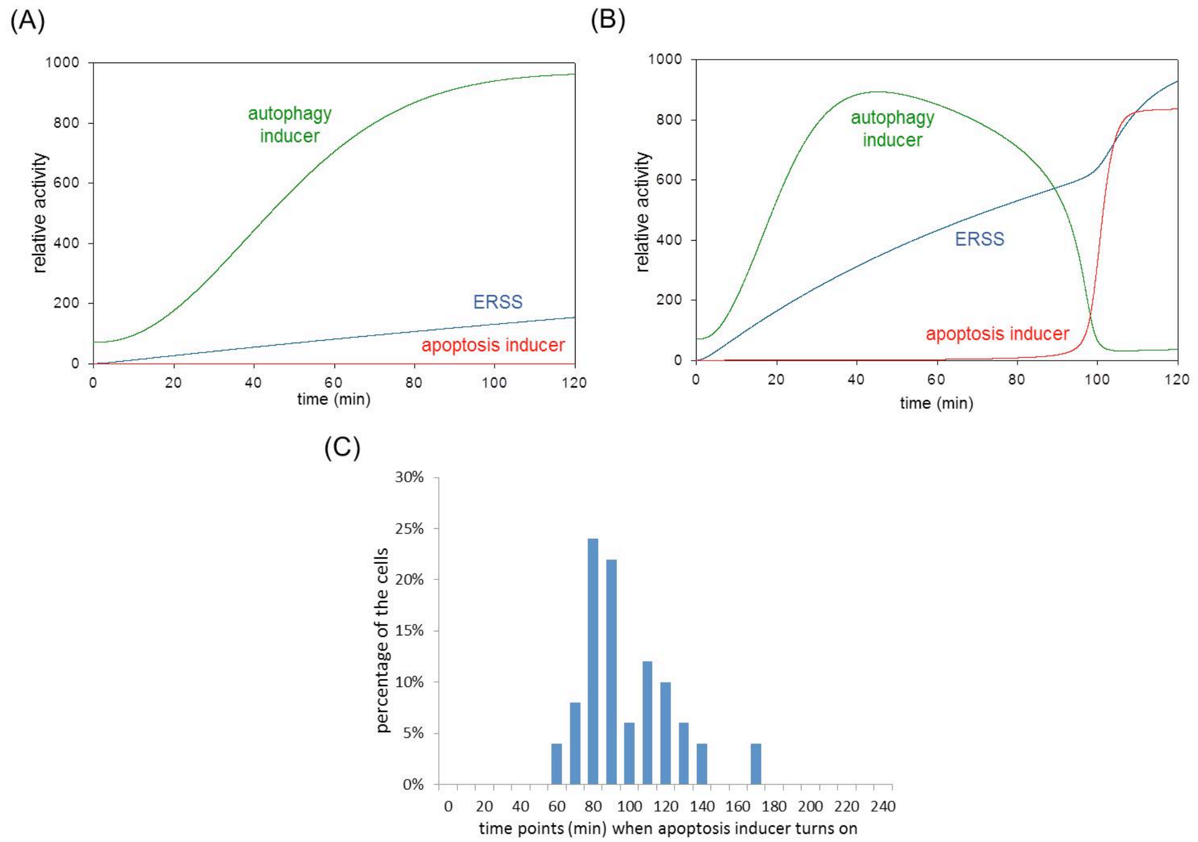

Figure S7. Mathematical analysis of autophagy-apoptosis crosstalk with respect to ER stress. The deterministic temporal dynamics is simulated for 120 min long treatment **(A)** with low (S0 = 1) and **(B)** with high (S0 = 10) level of ER stress. The dynamics of ER stress sensor, autophagy and apoptosis inducers are plotted. **(C)** The time distribution of apoptosis induction with respect to high level of ER stress 50 single cell simulations were done and measured the time points where apoptosis turned on (S0 = 10).

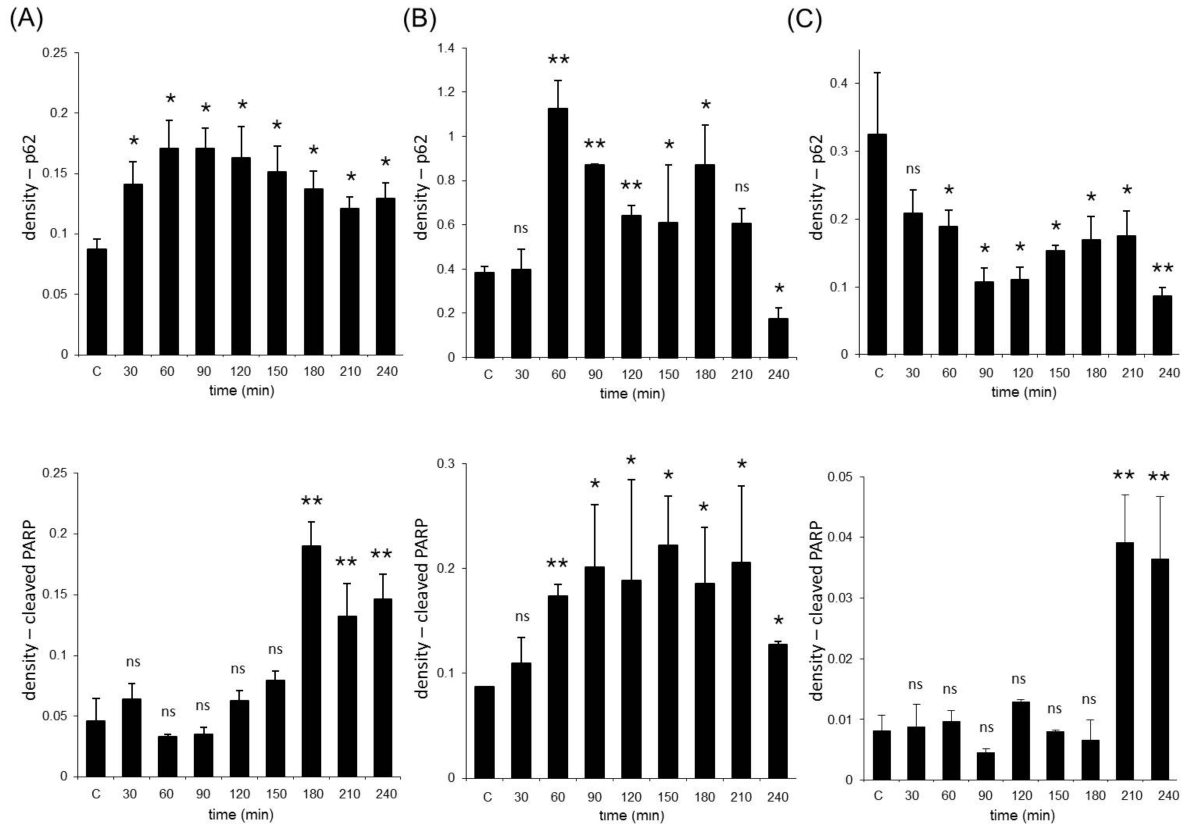

Figure S8. The role of autophagy to determine the activation threshold for apoptosis. HepG2 cells were pre-treated with autophagy inactivator (**(A)** and **(B)** 1 mM 3-methyladenine for 2 hours) or activator (**(C)** 100 μM metyrapone for 2 hours) before low (**(A)** 1 μM for 4 hours) or high (**(B)** and **(C)** 100 μM TM for 4 hours) level of TM treatment. Densitometry data represent the intensity of **(upper panel)** p62 and **(lower panel)** cleaved PARP bands. These band intensities are shown in arbitrary units after normalization of each band to its appropriate GAPDH band, separately (errors bars represent standard deviation, asterisks indicate statistically significant difference from the control: ⋆ - $p < 0.05$; ⋆⋆ - $p < 0.01$).

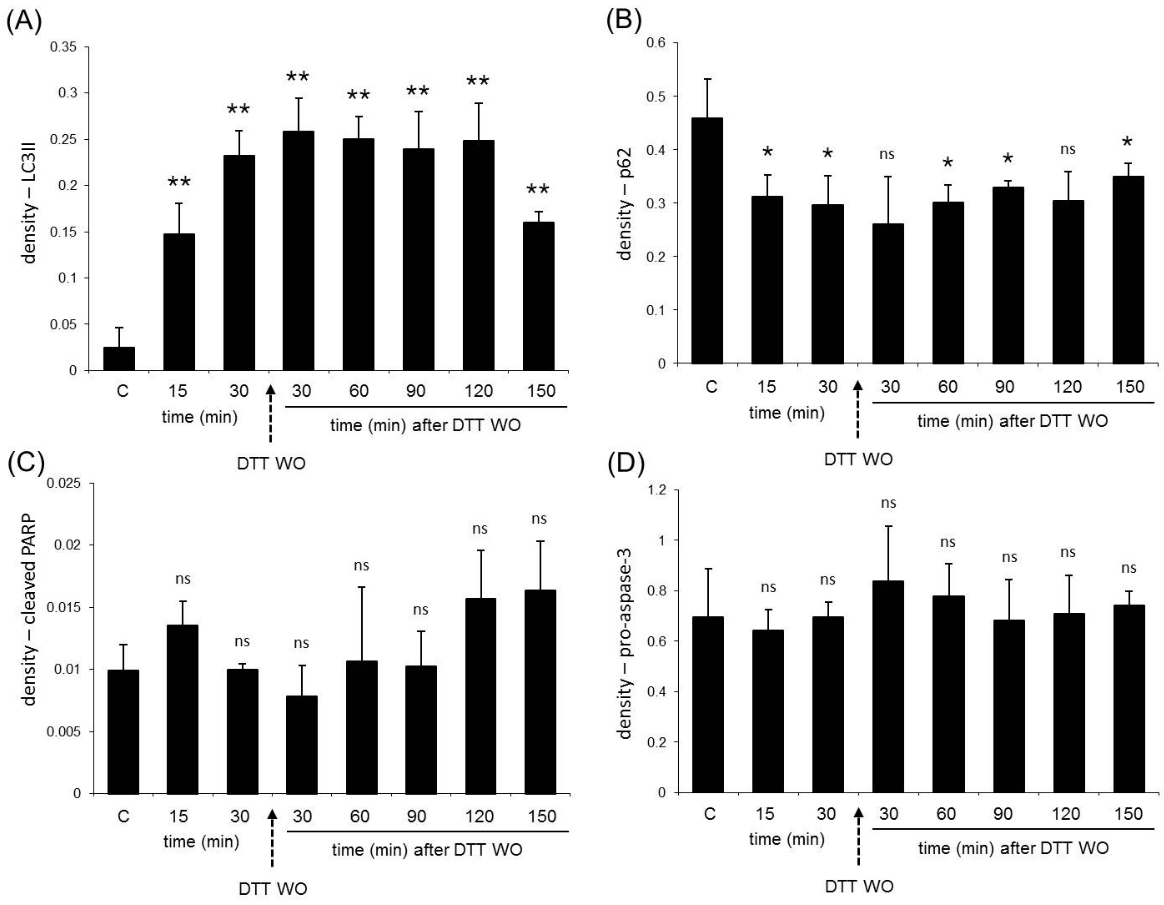

Figure S9. The effect of transient treatment with high level of ER stressor. HepG2 cells were treated with high concentration of DTT (10 mM) and washed out after 30 min. Densitometry data represent the intensity of **(A)** LC3II, **(B)** p62, **(C)** cleaved PARP and **(D)** procaspase-3 bands. These band intensities are shown in arbitrary units after normalization of each band to its appropriate GAPDH band, separately (errors bars represent standard deviation, asterisks indicate statistically significant difference from the control: ⋆ - $p < 0.05$; ⋆⋆ - $p < 0.01$).

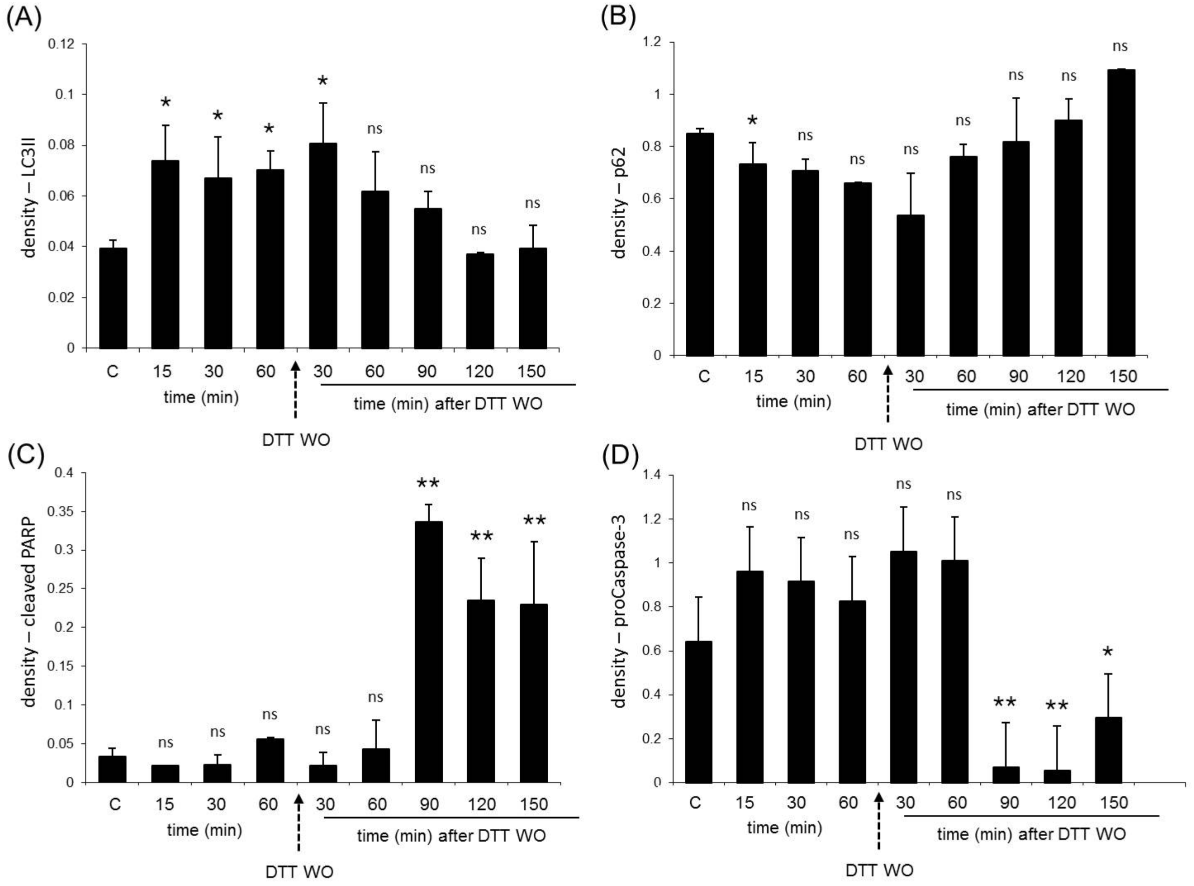

Figure S10. The effect of transient treatment with high level of ER stressor. HepG2 cells were treated with high concentration of DTT (10 mM) and washed out after 60 min. Densitometry data represent the intensity of **(A)** LC3II, **(B)** p62, **(C)** cleaved PARP and **(D)** procaspase-3 bands. These band intensities are shown in arbitrary units after normalization of each band to its appropriate GAPDH band, separately (errors bars represent standard deviation, asterisks indicate statistically significant difference from the control: ⋆ - $p < 0.05$; ⋆⋆ - $p < 0.01$).
